# Scientific Items

**Published:** 1879-05-20

**Authors:** 


### SCIENTIFIC ITEMS.

**Is Science Benevolent?**—Faraday had an idea, it is said, that it would be well if the secret of the composition of water were not discovered, as the power so gained might not be wisely used; and though the story may be nonsense, any power that, requiring skill and self-restraint for its use, was yet placed in the hand of all men would probably not be beneficial—would certainly not tend to that elevation in comfort which the popular mind permanently expects from science. Imagine the power of firing water discovered, made public from excellent motives, as in a patriotic war, and so becoming the property of a world in which one man in a thousand is probably a crypto-lunatic, anxious, above all things, for a supreme sensation. A discovery, quite possible, of the means of dissolving brick or stone within a definite area into pulp would materially interfere with the security of all property, as would for a time the realization of the Middle Age alchemists' dream. All these discoveries would, of course, to do mischief, require the aid of human malignity, in a consciously malignant state, but others are quite conceivable over which will would have no control. Suppose, for example, Sir G. Airey were to discover that a change had occurred in space, which within, say a century or two, would affect our universe, and inevitably draw the world out of its orbit, thereby pulverizing it to atoms; the effect of that discovery, fatal as it would be to foresight, to patriotism, to that long series of good impulses which have for their unconscious motor the belief that the human race will last, could be nothing but evil. Half the motives to energy and to self-restraint would disappear at once, while the temptation to use up the world, its forests, coal mines and resources generally, would be enormously exaggerated. Humanity would realize its mortality, and make the best—that is the worst—of its time. Not one of these suggestions, however, or many other much better ones which might be offered, will come in the least home to the minds of men taught by a few years' experience that science is kind, that knowledge is beneficial, and that every victory over the forces of nature tends to the comfort of man.—*The Spectator.*

**New Crater on the Moon.**—The reported appearance of a new crater on the moon was discussed at a meeting of the Royal Astronomical Society of London. Prof. Schmidt, of Athens, and other European observers, have noticed conditions of the lunar surface tending to confirm the alleged discovery by Klein; but there are some persons who think the same crater is shown on a photograph of the moon which was taken about ten years since. It was the opinion of most of the members, however, that the spot in this picture differed essentially from that which has lately been seen, and which is regarded as evidence that the moon is still subject to volcanic convulsions.—*Ex.*

**Electric Gas Lighting Apparatus.**—Mr. Wilson E. Facer, of Cleveland, Ohio, has devised an improved electric gas lighting apparatus which, by the simple opening and closing of an electric current, turns on and lights the gas or shuts it off. It is designed principally for lighting street lamps, but is capable of application to other purposes. —*Scientific American.*

**Opium Smoking.**—The Russian naturalist and explorer of New Guinea, Dr. Miclucho Maclay, has been trying an experiment upon himself in smoking opium. He secured the services of a friend to observe the effects. He smoked twenty-seven pipes in all—amounting to one hundred and seven grains of the drug. Though he had been fasting eighteen hours, the third pipe appeased all desire for food. After the fifth pipe he became sleepy, and after the thirteenth he was practically unconscious, although he talked and answered questions. A sleep of ten hours left him with an excellent appetite, but with a buzzing sensation and slight pain in his head. He experienced no dreams or visions of any kind whatever. The experiment was made in the Chinese Club at Hong Kong.—*Ex.*

**Cost of the Yellow Fever.**—Loss of life by yellow fever in the South last year is estimated at about 20,000 persons, and of money and trade at from $175,000,000 to $200,000,000—as great as the loss from the Chicago fire. But some good is likely to come out of this calamity. It is thought that henceforth quarantine regulations will be more thoroughly established than they have ever been. Apart from death and human suffering, negligence is the worst kind of political economy. Expenditure of one-twentieth part of what the fever has cost might have prevented it altogether.

**Air Purifier.**—A singular scheme for purifying the air of large towns has been proposed to the Society of Arts. It contemplates the erection of large and very lofty chimneys, to act as vast ventilators or air shafts for an entire city. Such a chimney would not only keep up a constant circulation of the air, but might carry off all the smoke of the town, and even the gas from the sewers as well. A modified and more practicable form of this plan is to provide a tall chimney for each block of houses, connecting all the smaller chimneys in the block with it.—*Ledger.*

**New Anæsthetic.**—A novel anæsthetic has been tried with great success in Paris by M. Paul Bert. It is a mixture of nitrous oxide and oxygen under tension. Under ordinary pressure the mixture has no anæsthetic effect, but under tension it produces entire insensibility. The pulse and complexion of the patient remain normal while under its influence, and there is no injurious reaction, the waking being calm and quiet.—*Ledger.*

**Astronomical Observations.**—There appears to be no doubt that the atmosphere at great elevations above the sea-level is best adapted for astronomical observations, so far as transparency is concerned; but according to Prof. S. P. Langley, of the Alleghany Observatory, Pa., there is no gain in steadiness. This conclusion is based upon his own experience in the West and on Mount Etna.—*Ledger.*

**Trichinæ Destroyed by Sulphurous Acid.**—An effective method of destroying trichinæ in pork has been discovered. The addition of a small proportion of sulphurous acid to the brine in which the pork is salted destroys the worms without injury to the meat. Such, says the Lancet, is the result of a number of experiments to test the relative value of different destructive agents.—*Ledger.*

